# Clinical marine biomedicine: An emerging area in clinical and translational medicine

**DOI:** 10.1002/ctm2.70303

**Published:** 2025-04-20

**Authors:** Xiaojun Yan, Wanxin Duan, Xiangdong Wang

**Affiliations:** ^1^ Department of Marine Biotechnology, School of Marine Science and Technology Zhejiang Ocean University Zhoushan China; ^2^ Department of Respiratory Critical Care Medicine Zhongshan Hospital Fudan University Shanghai Medical College Shanghai China; ^3^ Shanghai Institute of Clinical Bioinformatics Shanghai China; ^4^ Fudan University Center of Clinical Bioinformatics Shanghai China

**Keywords:** clinical, disease, human, marine biomedicine, oceanology

## Abstract

Marine biomedicine is an important field in oceanology and bio‐ecosystem and has evolved significantly alongside advances in biotechnology and growing understanding of marine life. In this perspective, we propose a refined concept of clinical marine biomedicine, with a clear mission to establish an emerging discipline that bridges marine biomedicine and clinical practice. The exploration of marine‐origin sources should be emphasised, with a strong focus on the identification, validation and development of human disease‐specific diagnostics and target‐oriented pharmaceutics. The perspective headlines some of critical components, including marine‐oriented human evolution and development, humanised marine‐based models, biomarker innovation and validation, marine microbiomes and metabolites, and target nutrition and therapy. We envision that clinical marine biomedicine will become a crucial pillar clinical molecular medicine, contributing to the improvement of human health and the prognosis of patient.

The biodiversities of marine organisms, biomes and biospheres have been recognised for centuries, with the scientific significance of marine biology receiving systematic attention since the year of 1950.^1^ The concept of *marine biomedicine* emerged as a novel interdisciplinary field within marine zoology in 1982,^2^ originating from one of the earliest marine biomedical research programs at the Medical University of South Carolina, led by Thomas C. Cheng. A decade later, Dr Kohl provided a comprehensive overview of the past, present and future of marine biomedicine conception, development and processing,^3^ including bioactive compounds from marine sources, synthetic analogues, marine‐derived pharmaceuticals, nutritional supplements, marine‐based biomaterials for medical applications and biomedical tools for disease monitoring and intervention. The scopes of marine biomedicine include oceanography, chemistry, ecology, physiology, anthropology, history, museology, pharmacology, phycology, theology, political science and beyond.^3^

With the rapid development of biotechnologies, molecular insights into the biodiversity of marine organisms are opening new avenues for the clinical application of marine biomedicine. These developments provide novel opportunities for the spatialisation and temporisation of marine discoveries, for the translation of marine‐derived innovations into clinical practices. Single‐cell transcriptomic profiling across different cell types, tissues, organs and species (including fishes) has enabled the construction of comprehensive cross‐species databases. These resources offer valuable information on molecular evolutions, developing biology, disease mechanisms, intercellular communications and gene regulatory networks.^4^, ^5^ Given these advances, the concept of *marine biomedicine* should be reconceptualised to reflect the evolving understanding of both marine and human biology. It should be redefined to encompass not only marine organisms and environments but also their relevance to human health and society, disease‐specific biomarkers and therapeutic targets. Therefore, we propose the term clinical marine biomedicine to describe this emerging field, which seeks to integrate marine‐derived biological insights into clinical and translational medicine. This Perspective headlights marine‐oriented human evolution and development, humanised marine‐based model, biomarker innovation and validation, marine microbiomes and metabolites, and target nutrition and therapy as the major parts of clinical marine biomedicine, to improve the quality of human health, the early detection of disease occurrence and the outcome of patient prognosis (Figure 1).

## 1 TRAJECTORY OF HUMAN EVOLUTION AND DEVELOPMENT

Recent studies have utilised marine sediment archives to investigate the complex relationship between climate change and human evolution, particularly over the past 4 million years.^6^ The genetic and molecular existence and evolution of marine organisms offers valuable insights into human biology, to predict potential therapy and inter‐influence between ocean and human health. The evolution trajectories of marine species can shape genetic adaptation mechanisms in humans, whereas anthropogenic activities are increasingly recognised as drivers of evolutionary changes within marine ecosystems. Additionally, the marine tetrapod can be reconstructed with labelled DNA and RNA sequencing, to trace the evolutionary history of marine invasion and reimagine the interaction between the marine and terrestrial life.

The trajectory of marine biology is particularly important in the maintenance of human health and in the balance between ocean and land environments. For instance, genome profiling of macroalgae reveals multicellular phenomes, genomic diversity and evolutionary mechanisms among diverse climates, phyla, locations, functionalities and evolutionary trajectories.^7^ Such molecular trajectories are especially valuable to be translated to assess the potential impacts of climate change, geographical variation and ecosystem shifts on human population evolution and health. Moreover, genomic profiles of the octopus have demonstrated that certain gene families, such as protocadherin involving in neuronal development and the C2H2 superfamily of zinc‐finger transcription factors, exhibit similarities to those found in vertebrates.^8^ Large‐scale genomic profiling and the identification of cephalopod‐specific gene rearrangements provide a model for predicting genome linkages, repetitive content, evolution of cephalopod morphological innovations, particularly those related to the nervous systems.

## 2 HUMANISED MARINE‐BASED MODEL

The zebrafish embryo, as one of the most recognised marine‐derived model organisms, has been extensively studied using single‐cell and special transcriptomic sequencing. These approaches have enabled the construction of valuable and fundamental spatiotemporal reference box with that capture gene regulatory networks and cellular interactions across developmental stages. Such spatiotemporal modules provide a multidimensional framework for understanding the molecular and cellular ecosystems involved in embryonic development.^9^ On basis of the spatiotemporal reference boxes, the model of human embryogenesis can be constructed to dynamically mirror the spatiotemporal evolution, development and trajectory of cell‐fate transitions, molecular changes and ligand–receptor distributions to better understand human development. A large number of obvious and latent variations of human evolution and development can be comprehensively investigated in zebrafish embryo, which can be hardly performed in human samples. A zebrafish single‐cell atlas of perturbed embryos has been constructed using single‐cell transcriptomic data from over 3 million cells, covering 1812 developing embryos across 19 dynamic time‐points and 23 genetic perturbations.^10^

## 3 BIOMARKER INNOVATION AND VALIDATION

Molecular representatives of marine vertebrates, organisms and plants are being explored to define their biodiversities at the level of genetics, transcriptomics and metabolomics, and to identify novel molecular biomarkers that can reflect aspects of human biology and pathology. This process is highly dependent upon the broadness of marine multi‐omics spectrum, the number of individual phyla and species, the strength of multi‐omics‐based reference library and the capacity of translational knowledge between clinical medicine and oceanology. Multi‐omics‐based functional reference boxes are critical for developing artificial‐intelligent driven single‐cell models.^11^ On the other hand, transcriptomic and proteomic profiles of fish immune systems can serve as analogues for human immunity, distinguishing resistant and susceptible genetics in parasite recognition and identifying disease‐specific target molecules like microRNA in the fish model infected with virus or bacteria. The specific biomarkers and molecular mechanisms of human embryogenesis are hardly investigated, especially those involved in spatiotemporal intercellular communications and regulations within specialised microenvironments, due to the limited accessibility to dynamic human samples.

Marine‐generated biological molecules hold significant potentials for the discovery and development of diagnostic biomarkers and therapeutic targets in human diseases. Their clinical values depend greatly upon the accuracy of biological function and the compatibility of species development. Alterations in marine microbial dysbiosis and dysfunction can be the potential biomarkers for bacterial infections, while metabolomic changes of marine plants and animals for virus infections. Similarly, microalgae can be employed as sensitive biosensors to detect environmental pollutants and assess associated health risks in human health. A cilia‐enriched interface cell state was uncovered during characterising the cell states, subtypes and interactions around the distinct borderline of the human melanoma boundary in the humanised zebrafish model of patient‐driven xenotransplantation using single‐cell and spatial transcriptomics.^12^ Such interface composed of specialised tumor and microenvironment cells was found as one of human melanoma characteristics. It implies that humanised zebrafish models can be translated into an efficient approach for clinical drug screening and evaluation, and into a disease‐specific tool for the clinical practice of precision medicine, like patient‐driven xenotransplantation models in other species.

## 4 MARINE MICROBIOMES AND METABOLITES

The immense amounts and species of marine microbiomes in the ocean ecosystem represent a valuable treasure for advancing our understanding of oceanography and uncovering of beneficial microbiota and metabolites for human. The vast diversities and novelties of marine microbiomes and their enzymes, metabolites and biosynthetic precursors, are being increasingly explored and discovered using multi‐/trans‐omics at the global scale. This exploration has become increasingly feasible since the spatiotemporal dynamics of marine microbiome function and morphology can now be tracked using single‐cell sequencing and multi‐omic profiling. The architecture and functioning of the ocean microbiome detected using population genomics may indicate and predict the patterns of population differentiation, adaptive mechanisms and genetic biodiversity, not only among marine microbial species, but also in comparisons between marine and human microbiomes, and in their varied responses to environmental chemicals and therapeutic agents.^13^ A deep understanding of ocean ecosystems by functional and population genomics provides a genetic blueprint and comprehensive database of oceanic microscopic organisms, enabling the screen and selection of novel bio‐therapeutic targets for human diseases. Among the newly identified biosynthetic gene clusters, the *phospeptin* and *pythonamide* pathways were identified and characterised in terms of their bioactive compound structure and enzymology, as one of microbiomics‐driven strategies for the discovery and development of natural products.^14^

## 5 TARGET NUTRITION AND THERAPY

A large number of marine organisms and corresponding metabolites exhibit various biological, immune‐modulatory and pharmacological activities, offering therapeutic potential against inflammation, cancer and aging in multiple formulations. For example, fucoidan‐related dietary supplements and nutraceuticals have been shown to regulate cell proliferation and death, and act as an adjuvant to clinical drugs in cancer treatment or to aid the recovery following chemotherapy, although the exact molecular mechanism remains unclear. Notably, fucoidan has recently been reported to promote effects of anti‐PD‐1 monoclonal antibody antitumor immunotherapy by rebalancing gut microbiota and its metabolites, improving the function of effector T cells and suppressing regulatory T cell production.^15^ Marine natural products, as efficient modulators, appear biological, nutritional and therapeutic effects, some of which have been clinically approved as first‐in‐class drugs of marine origin, while others remain in clinical trials. The pathway, time consume and investment from discovery to clinical application of marine‐origin drugs are highly dependent upon the structural complexity, drug‐ability, synthetic process and mechanism‐based declaration.^16^ It is concerning whether and how therapeutic effects of marine‐driven organisms, plants and metabolites can be defined and optimised in clinical precision medicine. One of the major challenges is to identify the bioactive molecules and their specific targets in human cells, for example, whether these molecules are directly derived from marine or aquatic sources, isolated as single active compounds or produced through bio‐ or chemo‐synthetic approaches.

In conclusion, we propose a redefinition of the field, from marine biomedicine to clinical marine biomedicine, with a clear mission to establish an emerging discipline and a translational channel from the understanding of marine biomedicine into clinical practices. The marine origin should be extensively explored, with a high focus on identification, validation and development of human disease‐specific diagnostics and target‐oriented pharmaceutics. The clinical marine biomedicine is consistent of marine‐oriented human evolution and development, humanised marine‐based models, biomarker innovation and validation, marine microbiomes and metabolites as well as targeted nutrition and therapy. We believe that clinical marine biomedicine will become an integral part of clinical molecular medicine and play important roles in improving the quality of human health and the prognosis of patient.

6

**FIGURE 1:**
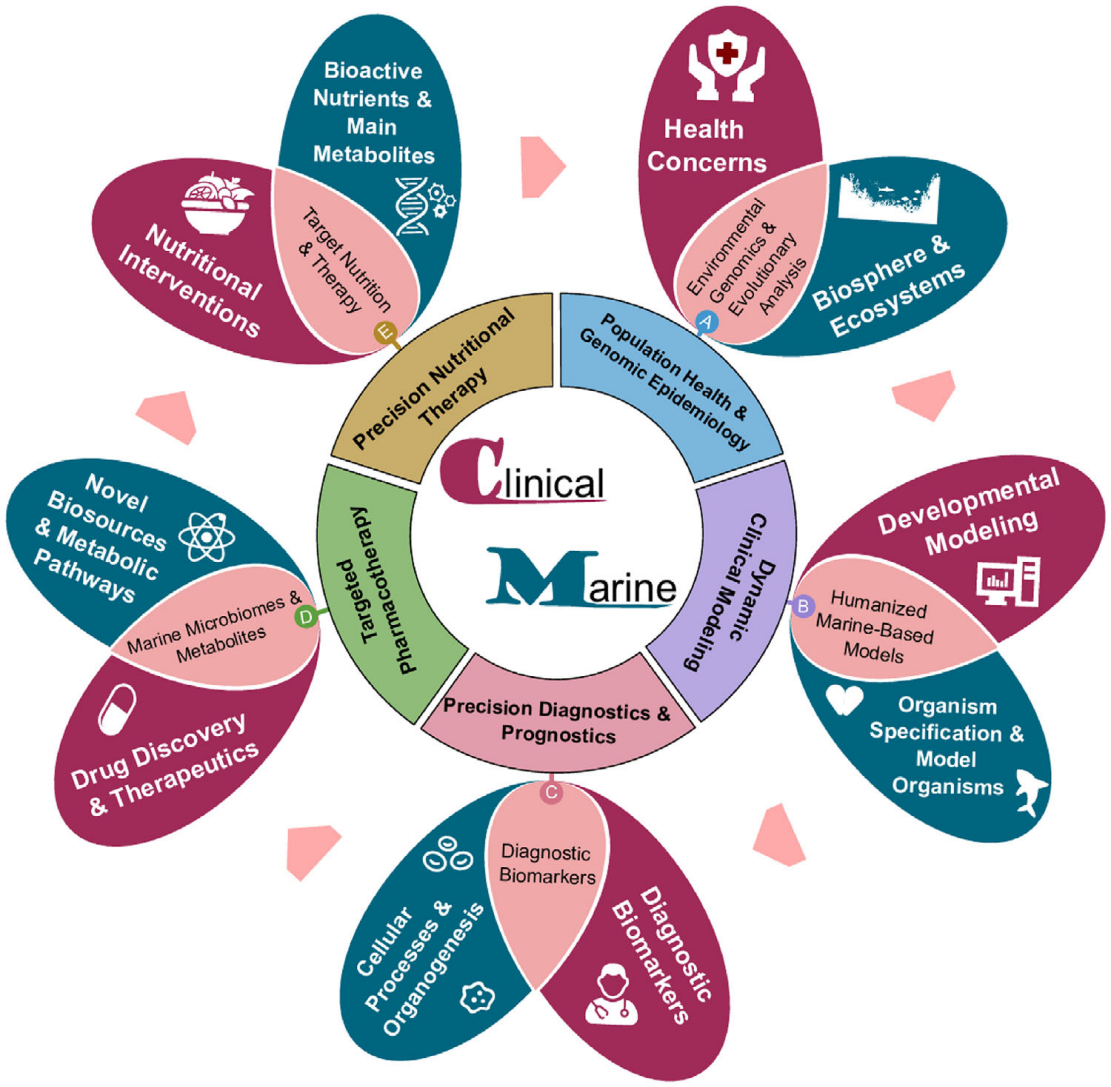
An integrated framework for interdisciplinary clinical marine biomedicine. This framework encompasses five emerging dimensions: (1) Population health and genomic epidemiology, which bridges marine biospheres and ecosystems for public health concerns; (2) dynamic clinical modelling, which integrates clinical developmental models with marine‐based model organisms; (3) precision diagnostics and prognostics, which decode marine‐specific cellular processes and organogenesis to facilitate clinical biomarker discovery; (4) targeted pharmacotherapy, which leverages novel bio‐sources and metabolic pathways to advance drug discovery and refined targeted pharmacotherapy; (5) precision nutritional therapy, which forms a population‐based nutritional integration between marine and clinical nutrients. The integration and translation strategy are illustrated as the crosstalk between the biomedicine (in red) and marine biology and biotechnology (in blue).
